# The *msaABCR* Operon Regulates Persister Formation by Modulating Energy Metabolism in *Staphylococcus aureus*

**DOI:** 10.3389/fmicb.2021.657753

**Published:** 2021-04-14

**Authors:** Shanti Pandey, Gyan S. Sahukhal, Mohamed O. Elasri

**Affiliations:** Center for Molecular and Cellular Biosciences, The University of Southern Mississippi, Hattiesburg, MS, United States

**Keywords:** *Staphylococcus aureus*, *msaABCR*, persister cells, ATP, membrane potential

## Abstract

*Staphylococcus aureus* is a major human pathogen that causes chronic, systemic infections, and the recalcitrance of these infections is mainly due to the presence of persister cells, which are a bacterial subpopulation that exhibits extreme, yet transient, antibiotic tolerance accompanied by a transient halt in growth. However, upon cessation of antibiotic treatment, a resumption in growth of persister cells causes recurrence of infections and treatment failure. Previously, we reported the involvement of *msaABCR* in several important staphylococcal phenotypes, including the formation of persister cells. Additionally, observations of the regulation of several metabolic genes by the *msaABCR* operon in transcriptomics and proteomics analyses have suggested its role in the metabolic activities of *S. aureus*. Given the importance of metabolism in persister formation as our starting point, in this study we demonstrated how the *msaABCR* operon regulates energy metabolism and subsequent antibiotic tolerance. We showed that deletion of the *msaABCR* operon results in increased tricarboxylic acid (TCA) cycle activity, accompanied by increased cellular ATP content and higher NADH content in *S. aureus* cells. We also showed that *msaABCR* (through MsaB) represses the *ccpE* and *ndh*_2_ genes, thereby regulating TCA cycle activity and the generation of membrane potential, respectively. Together, the observations from this study led to the conclusion that *msaABCR* operon deletion induces a metabolically hyperactive state, leading to decreased persister formation in *S. aureus*.

## Introduction

*Staphylococcus aureus* is a prominent human pathogen that causes systemic diseases, including infective endocarditis, osteomyelitis, bacteremia, and pneumonia (Lowy, 1998). Often chronic in nature, these infections are typically caused by device-associated biofilms that harbor persister cells (Lafleur et al., 2010; Lewis, 2010; Conlon et al., 2015). Persister cells are a distinct phenotypic variant in the bacterial population that exhibits transient, yet extreme, antibiotic tolerance without undergoing any genetic modification (Hobby, 1942; Bigger, 1944; Lewis, 2010). In the absence of antibiotic resistance, antibiotic-tolerant persister cells are believed to be largely responsible for the recalcitrance of bacterial infections and treatment failures (Conlon, 2014), thereby having high clinical significance. Indeed, the clinical relevance of *S. aureus* persister cells is now increasingly understood, as evidenced by their reported presence in recurring infections (Becker et al., 2014; Conlon, 2014). For instance, cases of bacteremia relapse, including recurring infections in endocarditis and in lung-transplanted patients, were caused by vancomycin-, linezolid-, and daptomycin-susceptible *S. aureus* (Ruiz et al., 2002; Corne et al., 2005; Welsh et al., 2011). Moreover, the clinical significance of *S. aureus* persister cells was also demonstrated in a chronic mouse wound infection model, in which these cells could not be eradicated by conventional drugs (Allison et al., 2011; Conlon et al., 2013). Indeed, persister cells are ubiquitous and have been studied in multiple bacterial species (Van den Bergh et al., 2017). However, the mechanism of persister formation has been a puzzling problem, particularly due to the transience of these cells and their small population size. Nonetheless, studies have so far demonstrated an association between the depletion of intracellular ATP and the formation of persister cells in major bacterial pathogens, including *S. aureus*, *Escherichia coli*, and *Pseudomonas aeruginosa* (Conlon et al., 2016; Shan et al., 2017; Cameron et al., 2018).

In recent years, an increasing number of studies have sought to understand the mechanism of persister formation, which is dependent on the metabolic activities of bacterial cells. Several studies have examined the contribution of metabolic genes and cellular ATP levels in the formation of *S. aureus* persister cells. For instance, Yee et al. (2015) identified the involvement of purine genes in persister cell formation in the presence of rifampicin. Likewise, Wang et al. (2015) showed that a mutant of succinate dehydrogenase (encoded by *sdhA*/*B*) is defective in formation of persister cells against an antibiotic (levofloxacin), heat, and oxidative stresses. Similarly, inactivation of *phoU* causes deficiency in the persister population due to increased metabolic activity (Shang et al., 2020). Recently, inactivation of tricarboxylic acid (TCA) cycle genes was shown to form drastically increased numbers of persister cells due to reduced ATP and membrane potential, suggesting the role of metabolism in persister formation (Wang et al., 2018; Zalis et al., 2019). This association was also evidenced in polymicrobial cultures in which *S. aureus* displayed increased antibiotic tolerance, accompanied by reduced membrane potential and intracellular ATP concentration (Nabb et al., 2019). The reduced ATP level associated with increased persister formation was also observed in inactivation of carbamoyl phosphate synthetase (encoded by *carB*) in the major pathogens *S. aureus*, *E. coli*, and *P. aeruginosa* (Cameron et al., 2018). In general, it appears that the conditions that reduce intracellular ATP and membrane potential, as well as cease transcription or translation, dramatically increase antibiotic tolerance in bacteria (Wood et al., 2013; Conlon et al., 2016; Shan et al., 2017). Together, these observations strongly suggest that the cellular metabolism and subsequent energy state of cells determine their switch to the persister phenotype.

Previously, we identified and characterized the *msaABCR* operon, constituting four genes (*msaA*, *msaB*, *msaC*, and anti-sense *msaR*) and regulated by two non-coding RNAs msaC and msaR (Sahukhal and Elasri, 2014). Further, we identified MsaB as a dual transcriptional regulator that functions as an activator as well as repressor to regulate its target genes (Batte et al., 2018; Pandey et al., 2019). In addition, Caballero and coworkers (Caballero et al., 2018) showed MsaB to be an RNA chaperone binding to 213 mRNAs, suggesting that MsaB is a global regulator. We have found a positive regulatory role for the *msaABCR* operon in biofilm development (Sahukhal et al., 2015), capsule production (Batte et al., 2018), antibiotic resistance (Bibek et al., 2019), antibiotic tolerance (Sahukhal et al., 2017), response to oxidative stress, and intracellular survival (Pandey et al., 2019). Moreover, in a recent study, we observed that an *msaABCR* operon mutant is attenuated in rat models of acute and chronic implant-associated osteomyelitis (Sahukhal et al., 2020).

Deletion of the *msaABCR* operon in the methicillin-resistant *S. aureus* (MRSA) strain USA300 LAC and the vancomycin-intermediate *S. aureus* (VISA) strain Mu50 formed fewer persister cells against several clinically relevant antibiotics (vancomycin, daptomycin, rifampicin, linezolid, and gentamicin). In transcriptomics and proteomics studies, we observed regulation of several metabolic genes by the *msaABCR* operon (Sahukhal et al., 2017). Together, these observations suggest a causal link between regulation of metabolic activities and formation of persister cells by the *msaABCR* operon. In the present study, we attempted to elucidate the role of the *msaABCR* operon in the regulation of metabolism and persister cell formation during the late stages of *S. aureus* growth.

## Materials and Methods

### Bacterial Strains, Culture Conditions, and Transposon Mutant Construction

Methicillin-resistant *S. aureus* USA300 LAC, isogenic *msaABCR* operon deletion mutant (△*msaABCR*), and △*msaABCR* + pCN34.*msaABCR* (Complementation) strains were used. The △*msaABCR* and △*msaABCR* + pCN34.*msaABCR* strains were generated as previously described (Sahukhal and Elasri, 2014). Briefly, the △*msaABCR* strain was generated by using pKOR1 allelic gene replacement method (Sahukhal and Elasri, 2014). The △*msaABCR* + pCN34.*msaABCR* complement strain was generated by inserting plasmid pCN34 that contained functional copy *msaABCR* operon gene in △*msaABCR* strain (Sahukhal and Elasri, 2014). Bacterial pre-cultures were prepared by inoculating cells from frozen culture into 5 ml of freshly prepared tryptic soy broth (TSB) (Becton, Dickinson and Company, Sparks, MD, United States) or chemically defined medium (CDM), as previously described (Batte et al., 2018). All cultures were incubated at 37°C with continuous shaking at 225 rpm. All the stationary-phase cultures were grown for 24 h, unless otherwise stated. All the transposon mutants used in this study were generated in the USA300 LAC background as previously described (Batte et al., 2018; Pandey et al., 2019). Briefly, a plasmid-cured derivative of LAC strain JE2 containing the transposon mutation within the coding regions of the corresponding genes was obtained from the Network on Antimicrobial Resistance in the *S. aureus* (NARSA) collection (BEI Resources). The mutation was then transduced into the USA300 LAC strain by generalized transduction using bacteriophage φ11. The transduction of the mutation into the recipient strains was confirmed by amplifying the beginning and end of the open reading frame of the corresponding genes, as previously described (Fey et al., 2013; Batte et al., 2018; Pandey et al., 2019). The detail information about the strains used in this study is listed in Supplementary Table 1.

### Measurement of ATP

Intracellular ATP content was measured using the BacTiter-Glo^TM^ kit according to the manufacturer’s instruction. Briefly, cells grown to the required phase were adjusted to an OD_600_ nm of 0.025 in 1 ml of TSB, from which 50 μl were dispensed into wells of an opaque 96-well plate, followed by the addition of an equal volume of BacTiter-Glo^TM^ Reagent. The luminescence was measured at OD_560_ nm after 5 min of incubation at room temperature.

### RNA Extraction, Reverse Transcription, and qRT-PCR

Aliquots of 500 μl from the required growth cultures were treated with equal volumes of RNAprotect bacterial reagent (Qiagen, Valencia, CA, United States) for 5 min at room temperature. Total RNA was extracted using the Qiagen RNeasy kit (Qiagen), and the quality and concentration of the extracted RNA were analyzed using a NanoDrop spectrophotometer (Thermo Scientific). Reverse transcription was performed with the iScript cDNA synthesis kit (Bio-Rad Laboratories, Hercules, CA, United States) using 1 μg of the total RNA isolated according to the manufacturer’s instruction. Next, qRT-PCR was performed using SYBR Green supermix (Bio-Rad), and the relative fold change in gene expression was calculated using *gyrB* as an endogenous control gene. The data represent the results from three independent experiments. All primers used in qRT-PCR are listed in Supplementary Table 2.

### Measurement of NAD^+^/NADH Content

Overnight cultures were diluted to an OD_600_ of 0.05 and grown until late exponential phase (OD_600_ of 4.0). Cells were harvested and washed twice with ice-cold phosphate-buffered saline (PBS), and NADH levels were measured according to the manufacturer’s instructions (NAD^+^/NADH quantitation kit, Sigma-Aldrich). Values represent the absolute concentrations of NAD^+^ and NADH per μg of protein.

### Persister Assays

Frozen *S. aureus* cells were inoculated in fresh TSB and grown for ∼3 h. These pre-cultures were normalized to an OD_600_ nm of 0.05 in 5 ml of TSB at a flask-to-medium ratio of 10:1 and incubated at 37°C until the required growth phase. Persister assays in late exponential phase were performed when the culture OD_600_ reached 4.0 (after 3.5–4 h growth), whereas the culture was grown for 24 h to reach stationary phase. When the cells reached the required growth phase, 100-μl aliquots were diluted in PBS and plated for the initial colony-forming unit (CFU) counting, whereas 3 ml of the culture was transferred to a 50-ml tube and was individually challenged with gentamicin (20 μg/ml, 20 × MIC), vancomycin (62.5 μg/ml, 100 × MIC), ciprofloxacin (250 μg/ml, 100 × MIC), or tobramycin (20 μg/ml, 20 × MIC). The cultures were then incubated to determine the persister count. At the designated time post exposure, 100 μl of culture was removed, washed with PBS, diluted, and plated for CFU counting.

For the persister assay of biofilms, a previously described *in vitro* catheter model of biofilm formation was implemented (Weiss et al., 2009; Sahukhal et al., 2017). Briefly, 1-cm-long sterile catheters (Becton Dickinson Infusion Therapy System Inc., Sandy, UT, United States) were incubated with 20% human plasma (Sigma-Aldrich, Saint Louis, MO, United States) overnight in 24-well microtiter plates. After plasma coating, the catheters were incubated with *S. aureus* cells (OD_600_ of 0.05) in biofilm medium (TSB + 0.5% glucose + 3% NaCl) for 24 h with continuous shaking. After incubation, the catheters were removed, washed gently with PBS to remove the non-adherent *S. aureus* cells, and sonicated for initial CFU counting. The remaining catheters were treated with tobramycin (20 μg/ml, 20 × MIC) and further incubated at 37°C. Each day after treatment, the catheters (*n* = 3) were recovered, and the spent medium was replaced with fresh biofilm medium (500 μl) with 20 μg/ml tobramycin. The catheters were gently rinsed in PBS to remove any non-adherent bacteria, placed in glass tubes, and sonicated to recover the adherent *S. aureus* cells. The recovered CFUs were counted for all three catheters. This procedure was continued for 4 days. All experiments were performed in triplicate and repeated twice. For the persister cell count in the presence of arsenate and carbonyl cyanide m-chlorophenyl hydrazone (CCCP), stationary-phase cells were challenged with arsenate (1 mM) and different concentrations of CCCP (0.001–10 μM) for 30 min prior to the addition of gentamicin. At 24 h post exposure, the cells were harvested, washed, and plated for CFU counting. All experiments were performed with at least three biological replicates. MIC values for the antibiotics are listed in Supplementary Table 3.

### Measurement of Membrane Potential

Membrane potential was assessed using the BacLight^TM^ Bacterial Membrane Potential kit (Life Technologies, Carlsbad, CA, United States). Stationary-phase cells were washed and resuspended with 0.5 ml of filtered PBS (2 × 10^7^ cells). Next, 16.6 μl of the fluorescent membrane potential indicator dye 3, 3′-diethyloxacarbocyanine iodide (DiOC_2_(3)) was added and incubated at room temperature for 30 min. The fluorescent signals of 100,000 cells were recorded using a Fortressa flow cytometer (BD Biosciences, San Jose, CA, United States). Negative control cells were incubated with 10 μM CCCP for 30 min prior to adding DiOC_2_(3). The ratio between red fluorescence (PE-A) and green fluorescence (FITC) was calculated and analyzed with FlowJo v10 software.

### Expression and Purification of MsaB Protein

The MsaB protein was expressed in △*msaABCR* of USA300 LAC strain as previously described (Pandey et al., 2019). Expression of protein (MsaB-His) was induced with the addition of 10 μM cadmium chloride (CdCl_2_) at the exponential phase (OD_600_ of 0.5–0.7) followed by incubation further for ∼4 h with continuous shaking. After 4 h of induction the cells were pelleted, resuspended in PBS (pH 7.4) with a protease inhibitor cocktail, and then lysed by bead beating and sonication. The cell lysate was then centrifuged at 10,000 × *g* for 30 min to remove the cell debris. The 6× His-MsaB fusion protein was purified from the clear lysate using nickel column (HisPur Ni-nitrilotriacetic acid [Ni-NTA] resin; Thermo Scientific) method.

### Electrophoretic Mobility Shift Assay (EMSA)

Electrophoretic mobility shift assays (EMSAs) were performed using the LightShift^TM^ optimization and control kit (Thermo Scientific) according to the manufacturer’s protocol and as previously described (Batte et al., 2016; Pandey et al., 2019). Briefly, the binding reaction mixture was prepared with ultrapure water containing 1× binding buffer, 50 ng poly (dI-dC), 2.5% (v/v) glycerol, 0.05% (v/v) NP-40, and 5 mM MgCl_2_. Biotin-labeled single-stranded DNA (ssDNA) upstream of the transcription initiation site of the *ndh*_2_ gene and double-stranded DNA (dsDNA) of the *ccpE* gene in the appropriate concentrations were incubated with increasing concentrations of purified recombinant MsaB–His protein in the binding reaction buffer and unlabeled specific probe when required. The mixture was then incubated at room temperature for 25 min and subjected to electrophoresis at 100 V for 1 h in a pre-run 5% Tris-borate EDTA (TBE) gel. The samples were then transferred to a nylon membrane, incubated for 45 min in the cold, crosslinked, and processed for the detection of samples. The protein–DNA complexes in the gel were then visualized using the Chemiluminescent EMSA kit (Thermo Scientific) according to the manufacturer’s protocol and imaged with a ChemiDoc system (Bio-Rad). The probes used for EMSA studies are listed in Supplementary Table 2.

### Gentamicin Uptake Assay

Gentamicin–Texas Red conjugate (Gent: TR) was prepared as previously described (Dai et al., 2006) with slight modifications. Briefly, 440 μl of 50 mg gentamicin sulfate ml^–1^ was mixed with 60 μl of 2 mg amine-reactive Texas Red-X succinimidyl ester (Invitrogen) ml^–1^ in anhydrous *N*, *N*-dimethylformamide. The mixture was gently rotated for 3 days at 4°C to produce an approximately 30: 1 molar ratio (∼10 mM gentamicin and 0.3 mM Texas Red reagent) of Gen: TR. Next, 100 μl of the Gen: TR was added to the stationary phase cells (2 × 10^7^), washed, and resuspended in 2 ml PBS for 3 h at 37°C with continuous rotation. After incubation, the cells were washed twice, resuspended in 500 μl PBS, and evaluated by flow cytometry with excitation/emission maxima at 595/615 nm.

### Statistical Analyses

All data were analyzed using GraphPad Prism (version 8) software using student’s *t*-test (unpaired) or one-way analysis of variance (ANOVA) followed by Tukey’s multiple comparison test, with *P* < 0.05 considered as statistically significant.

## Results

### The *msaABCR* Operon Regulates Energy Metabolism in *S. aureus*

**FIGURE 1:**
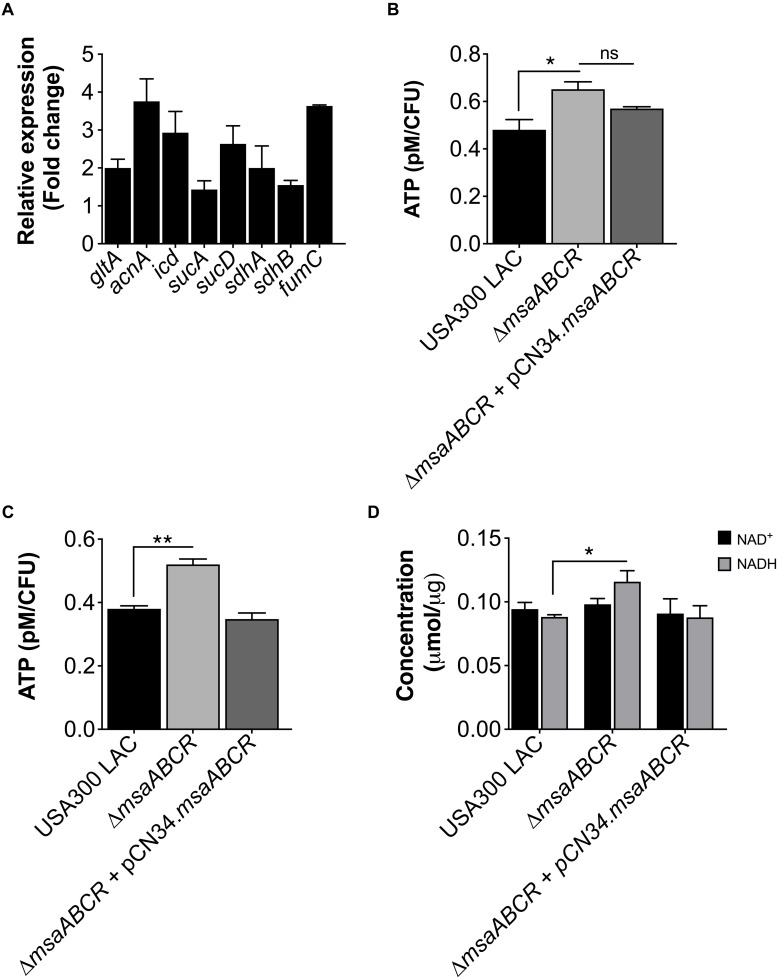
Measurement of TCA cycle activity, intracellular ATP level, and NAD^+^/NADH content. **(A)** Fold change expression of TCA genes (*gltA*, *acnA*, *icd*, *sucA*, *sucD*, *sdhA/B*, and *fumC*) in late exponential growth phase for the △*msaABCR* compared with the USA300 LAC strain, as measured by qRT-PCR. **(B)** The ATP concentrations per CFU of all strains were measured in late exponential and **(C)** stationary growth phases. The data represent the average of three ATP concentrations per CFU for each individual sample measured with two biological replicates. **(D)** Absolute concentration of NAD^+^/NADH content in all strains grown to late exponential phase. Error bars represent the standard error of the mean (SEM) for three independent experiments. Statistical significance was determined using analysis of variance (ANOVA) followed by Tukey’s multiple comparison test. *, *P* ≤ 0.05; **, *P* ≤ 0.001.

Based upon previous work from our laboratory (Sahukhal et al., 2017), we hypothesized that the deletion of the *msaABCR* operon in *S. aureus* cells would result in increased metabolic activity, thus inducing a higher energy state that prevents persister cell formation. To test this prediction, we first measured the expression of TCA genes and ATP levels in our test strains (USA300 LAC, △*msaABCR*, and △*msaABCR* + pCN34.*msaABCR*). In *S. aureus*, the TCA cycle is activated during late exponential growth phase to generate ATP when glucose is exhausted (Somerville et al., 2002; Somerville, 2016). We examined TCA cycle activity by measuring the expression of TCA genes (*gltA*, *acnA*, *icd*, *sucA*, *sucD*, *sdhA/B*, and *fumC*) in late exponential growth phase via quantitative real-time PCR (qRT-PCR). As expected, we found transcriptional upregulation of all the TCA genes in the △*msaABCR* compared with the USA300 LAC strain (Figure 1A). Likewise, the △*msaABCR* exhibited higher ATP levels relative to the USA300 LAC strain in the late stages of exponential growth, including stationary growth phase (Figures 1B,C). Considering that the TCA cycle provides reduced dinucleotides, we measured NADH content in our strains, and the results showed significantly higher NADH content in the △*msaABCR* than in the USA300 LAC strain (Figure 1D).

**FIGURE 2:**
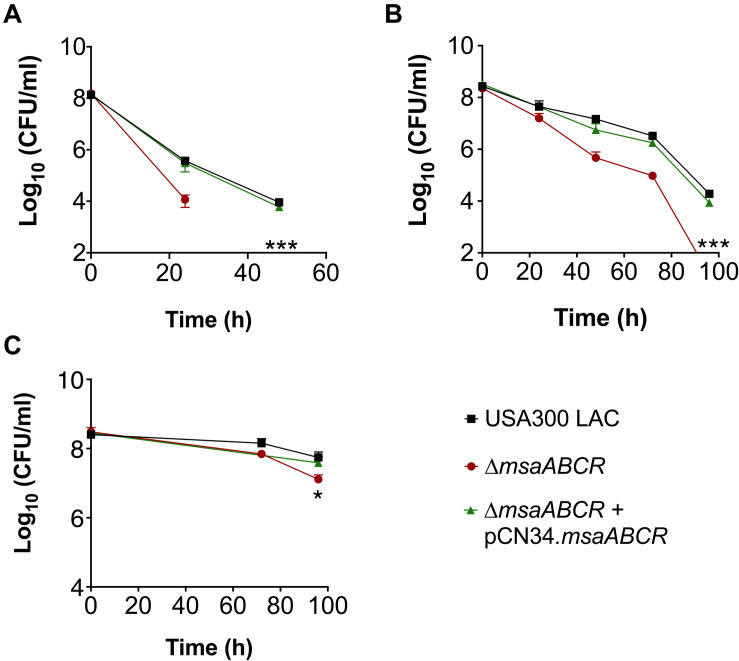
Persister assay in late exponential growth phase. **(A–C)** The killing of USA300 LAC, △*msaABCR*, and △*msaABCR* + pCN34.*msaABCR* cells in the presence of antibiotics at the indicated times. All strains were grown in TSB to late exponential growth phase (OD_600_ of 4.0) and exposed to gentamicin (20 × MIC), vancomycin (100 × MIC), or ciprofloxacin (100 × MIC). **(A)** Gentamicin treatment. **(B)** Vancomycin treatment. **(C)** Ciprofloxacin treatment. Error bars represent the standard error of the mean (SEM) from three independent experiments. Statistical significance was determined using ANOVA followed by Tukey’s multiple comparison tests. *, *P* < 0.05; ***, *P* < 0.0005.

Since we observed increased TCA cycle activity and intracellular ATP level in the △*msaABCR*, we hypothesized that this increased energy level would lead to decreased persister formation. To test this prediction, we first measured the persister fraction in late exponential growth phase in the presence of antibiotics targeting protein synthesis (gentamicin), cell wall synthesis (vancomycin), and replication (ciprofloxacin). In this growth phase, the △*msaABCR* cells were eradicated by gentamicin after 24 h, whereas the USA300 LAC and △*msaABCR* + pCN34.*msaABCR* strains showed prolonged survival for up to 48 h, with corresponding biphasic killing curves (Figure 2A). Likewise, the △*msaABCR* showed significantly fewer persister cells than the USA300 LAC and △*msaABCR* + pCN34.*msaABCR* strains when treated with vancomycin and ciprofloxacin (Figures 2B,C). Interestingly, all the test strains showed a higher fraction of persister cells in the presence of ciprofloxacin than with gentamicin or vancomycin.

**FIGURE 3:**
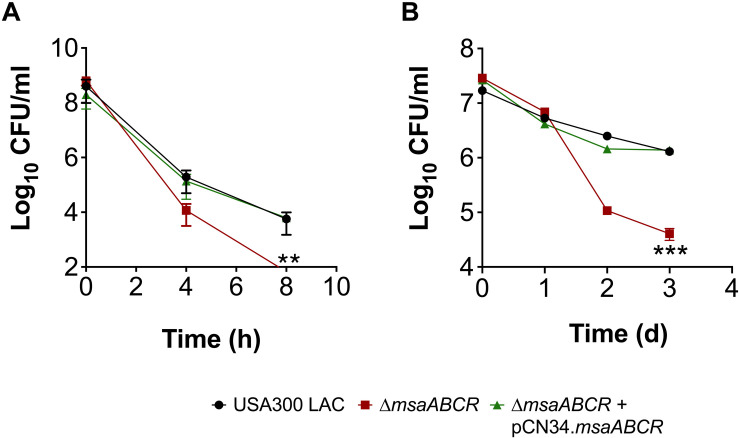
Measurement of persister cells in the presence of aminoglycoside stress. **(A,B)** The killing of USA300 LAC, △*msaABCR*, and △*msaABCR* + pCN34.*msaABCR* cells in the presence of tobramycin at the indicated time points. Strains grown to stationary phase and on biofilms were exposed to tobramycin (20 × MIC). **(A)** Tobramycin treatment in stationary-phase culture. **(B)** Tobramycin treatment on catheter biofilms. Error bars indicate SEM of at least three independent experiments. Statistical significance was determined using ANOVA followed by Tukey’s multiple comparison tests. **, *P* < 0.001; ***, *P* < 0.0005.

Given that the antibiotic tolerance of persister cells is associated with slow growth (dormancy), our previous study ruled out the possibility that defective persister cell formation in the △*msaABCR* was influenced by a difference in growth rate and yield in the stationary phase (Sahukhal et al., 2017). In our previous study, we observed that the △*msaABCR* failed to form persister cells in stationary growth phase in the presence of gentamicin. To further examine the specificity of aminoglycoside, in this study, we measured the persister fraction in the presence of tobramycin. In both stationary-phase and biofilm growth conditions, the △*msaABCR* formed significantly fewer persister cells than the USA300 LAC and △*msaABCR* + pCN34.*msaABCR* strains (Figures 3A,B). Altogether, these results suggested that the role of *msaABCR* in persister formation is specific to aminoglycoside stress in the stationary growth phase.

**FIGURE 4:**
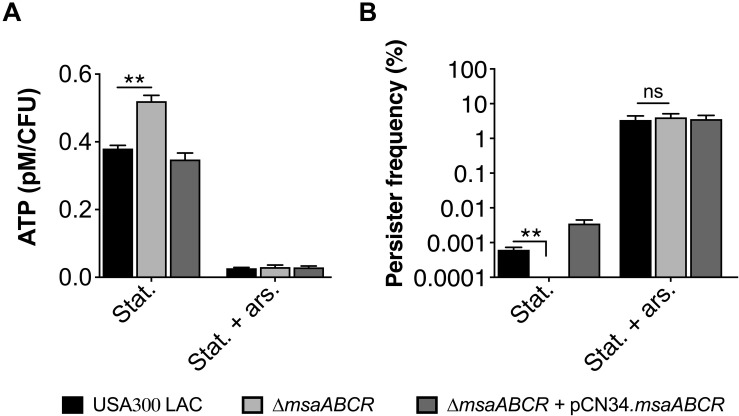
Measurement of ATP level and persister frequency in the presence of arsenate. **(A)** ATP concentrations were measured at stationary growth phase in the presence of 1 mM arsenate. The data represent the average of three ATP concentrations per CFU of each individual sample measured with two biological replicates. **(B)** Persister frequency (log_10_ survival fraction) was measured in stationary-phase culture against gentamicin (20 μg/ml), with and without prior exposure to arsenate. The data represent the results from three independent experiments. Error bars represent the SEM. Statistical significance was determined using ANOVA followed by Tukey’s multiple comparison tests. **, *P* < 0.001.

Several studies have reported increased persister formation corresponding to the depletion of ATP in different bacterial species (Conlon et al., 2016; Shan et al., 2017). These studies, as well as our observations in late exponential phase, suggest a causal link between high intracellular ATP content and decreased persister formation in *S. aureus* cells. To further examine the importance of ATP in antibiotic tolerance, we measured the persister fraction by depleting intracellular ATP with arsenate, which lowers the rate of ATP synthesis by forming hydrolyzable ADPs. When cell cultures were pre-exposed to 1 mM arsenate, the intracellular ATP level was reduced, resulting in a dramatic increase in the persister level in the △*msaABCR* compared with the USA300 LAC and △*msaABCR* + pCN34.*msaABCR* strains (Figures 4A,B). These results together demonstrate that a high cellular energy level is necessary for the killing efficiency of bactericidal antibiotics and that a low energy level increases antibiotic tolerance in bacterial cells.

**FIGURE 5:**
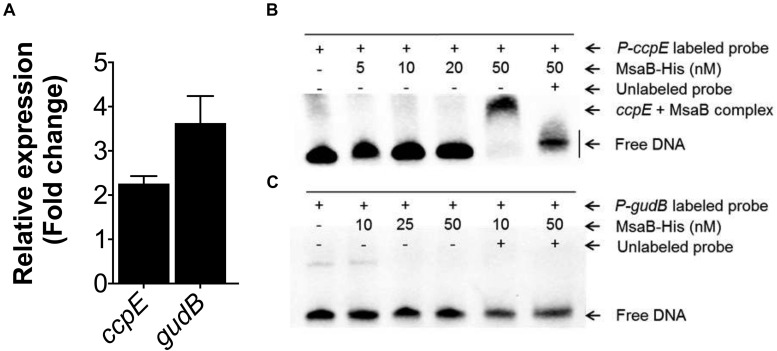
Measurement of gene expression and electrophoretic mobility shift assay (EMSA). **(A)** Fold change expression of *ccpE* in late exponential growth phase and *gudB* in the chemically defined medium (CDM) for the △*msaABCR* compared with the USA300 LAC strain, as measured by qRT-PCR. **(B)** MsaB binds to the *ccpE* promoter. Increasing concentrations of MsaB–His protein were incubated with a biotin-labeled, 50-bp, dsDNA probe incorporating the promoter region of *ccpE*. The band shift is shown in lane 5, while the shift was reversed in a competition assay with a 100-fold greater concentration of unlabeled probe in lane 6. **(C)** MsaB does not bind to the promoter of *gudB* as evidenced by the absence of band shift.

Next, we sought to examine *msaABCR* regulation of the TCA cycle. The LysR-type regulator catabolite control protein (CcpE) functions as a major positive transcriptional regulator of aconitase, encoded by the first gene in the TCA cycle (*acnA*) (Hartmann et al., 2013). To delineate the regulatory role of the *msaABCR* operon, we measured the expression of *ccpE* in late exponential growth phase. qRT-PCR revealed upregulation of *ccpE* (>2-fold) in the △*msaABCR* compared with the USA300 LAC strain (Figure 5A). Likewise, in the late stages of *S. aureus* growth, catabolism of the amino acid glutamate by glutamate dehydrogenase (encoded by *gudB*) ultimately fuels the TCA cycle through 2-oxoglutarate. Further, to observe the specific requirement of amino acid catabolism, we measured the expression of *gudB* in our test strains grown in chemically defined media (CDM) containing amino acids. The qRT-PCR result showed increased expression (>3-fold) of *gudB* in the △*msaABCR* compared with USA300 LAC strain (Figure 5A). Further, to examine MsaB regulation of *ccpE* and *gudB*, we performed an EMSA, which showed MsaB binding to the *ccpE* promoter (Figure 5B) and thereby suggested that MsaB regulates the TCA cycle via *ccpE*. This also confirms our previous finding of MsaB regulation of *ccpE* in the UAMS-1 strain (Batte et al., 2018). However, the absence of binding to the promoter of *gudB* suggests that its regulation by MsaB might be indirect (Figure 5C). All these observations lead us to conclude that MsaB directly regulates *ccpE* to control TCA cycle activity and that the increased energy state in the △*msaABCR* led to decreased persister cell formation in late exponential growth phase.

### Decreased Persister Formation in the Stationary-Phase △*msaABCR* Is Dependent on Increased Membrane Potential

**FIGURE 6:**
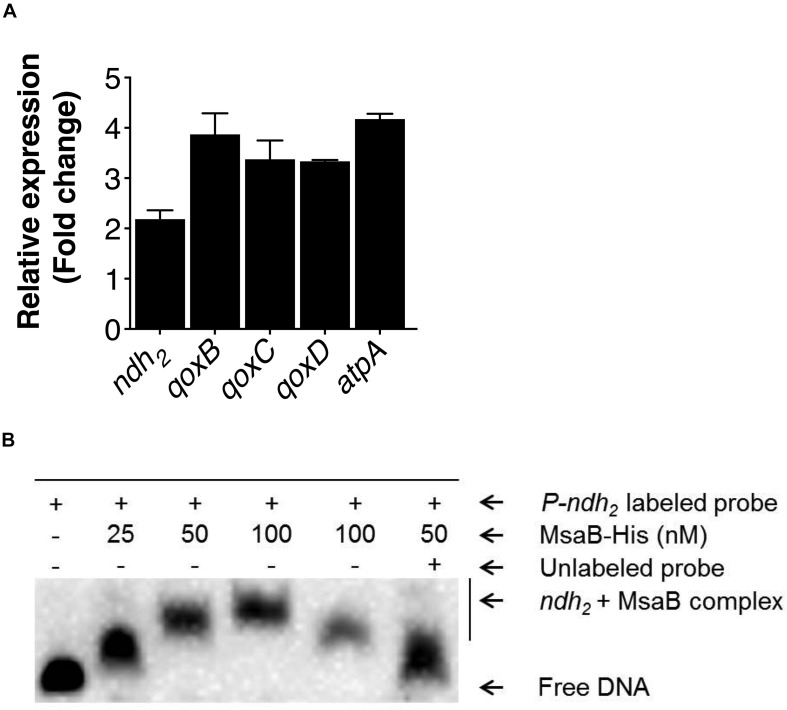
Measurement of electron transport chain (ETC) gene expression and regulation of *ndh*_2_. **(A)** Expression of ETC genes as well as *gudB* was measured by qRT-PCR of RNA samples isolated from cells grown overnight in chemically defined medium (CDM). The bar graph shows the fold change in expression of genes in the △*msaABCR* compared with the isogenic USA300 LAC strain. **(B)** EMSA shows MsaB binding to the promoter of *ndh*_2_. Increasing concentrations of MsaB were incubated with appropriate concentrations of ssDNA from the region upstream of the transcription initiation site for *ndh*_2_. The band shift was reduced in a competition assay with a 100-fold greater concentration of unlabeled *ndh*_2_ probe.

The activated TCA cycle generates large amounts of reduced dinucleotides, the oxidation of which requires the electron transport chain (ETC) (Somerville et al., 2002; Somerville and Proctor, 2009). The electrons entering into the ETC from NADH are transferred to menaquinone by the NADH dehydrogenase complex, and through a series of oxidation reactions in cytochrome oxidases, finally produces water and pumps protons (H^+^) across the membrane to produce pH and electrochemical gradients or membrane potential (Δψ). In *S. aureus*, the NADH dehydrogenase II (SAUSA300_0844, encoded by *ndh*_2_) and cytochrome aa_3_ oxidase (encoded by *qoxBCD*) contribute to the generation of membrane potential (Clements et al., 1999; Friedrich Götz, 2013; Hammer et al., 2013; Mayer et al., 2015). Likewise, the F1F0 ATPase (encoded by *atpA*) also contributes to the generation of proton-motive force (PMF) by pumping out protons upon ATP hydrolysis (Grosser et al., 2018; Wang et al., 2018). In the stationary growth phase, the TCA cycle is de-repressed when amino acids are catabolized. Therefore, we measured the relative expression of genes involved in the ETC, including *ndh*_2_, *qoxBCD*, and *atpA*, in the test strains grown in CDM containing amino acids. The qRT-PCR results showed a significant increase (>2-fold) in expression of the *ndh*_2_, *qoxBCD*, and *atpA* genes in the △*msaABCR* compared with the USA300 LAC strain (Figure 6A). A previous study reported that *ndh*_2_ is a major NADH: quinone oxidoreductase, disruption of which showed a large reduction (70%) in membrane potential compared with the wild-type USA300 strain (Mayer et al., 2015). Considering the importance of *ndh*_2_ in the generation of membrane potential, we sought to investigate its regulation by MsaB. We performed EMSA to examine the binding of MsaB to the promoter region of *ndh*_2_. The result showed MsaB binding to ssDNA upstream of the transcription initiation site for *ndh*_2_ (Figure 6B). However, MsaB did not show binding to the same region when using dsDNA. This result suggests that MsaB regulates *ndh*_2_, either at the post-transcriptional level or as an RNA chaperone, as previously reported (Caballero et al., 2018). Together, these observations suggest that *msaABCR* (MsaB) directly regulates NADH oxidation via negative regulation of *ndh*_2_ in the USA300 LAC strain.

**FIGURE 7:**
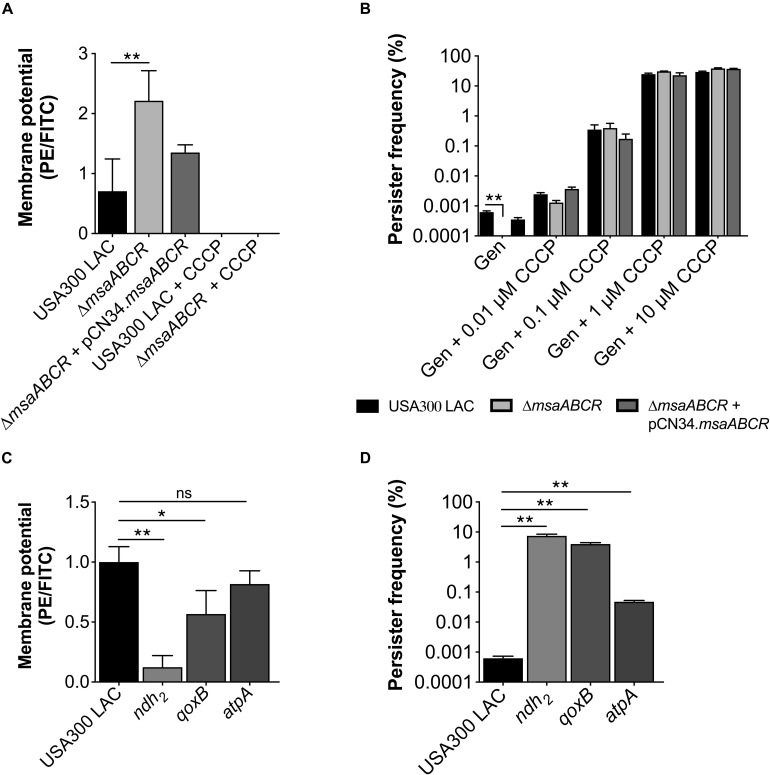
Measurements of membrane potential and persister frequency. **(A)** Stationary-phase cells washed twice with PBS were incubated with 3, 3′-diethyloxacarbocyanine iodide (DiOC_2_(3)) for 30 min in room temperature. After incubation, the cells were subjected to flow cytometry. The data represents the ratio between channel F3 (red fluorescence) and F1 (green fluorescence), calculated with FlowJo software. **(B)** Stationary-phase cells pre-incubated for 30 min with different concentrations of carbonyl cyanide m-chlorophenyl hydrazone (CCCP) were exposed to gentamicin (20 μg/ml). At 24 h post exposure, the cells were harvested, washed, and plated for CFU counting. **(C)** Measurement of membrane potential. **(D)** Measurement of persister fraction in the mutants of the ETC genes *ndh2*, *qoxB*, and *atpA* compared with the isogenic USA300 LAC strain in the presence of gentamicin (20 μg/ml). Data represents the log_10_ survival fraction for the average of three independent experiments. Error bars represent SEM. Statistical significance was determined using ANOVA followed by Tukey’s multiple comparison tests. *, *P* < 0.05; **, *P* < 0.001.

Since we observed upregulation of ETC genes in the △*msaABCR* that contribute to membrane potential, we measured the membrane potential of the mutant via flow cytometry using the fluorescent membrane stain DiOC_2_ (3) and compared with the USA300 LAC strain. We also measured the membrane potential of the *ndh*_2_, *qoxB*, and *atpA* mutants. The results showed higher membrane potential in the △*msaABCR* than in the USA300 LAC strain (Figure 7A). To further confirm the contribution of membrane potential in persister generation, we measured the persister fraction in the presence of the proton ionophore carbonyl cyanide m-chlorophenyl hydrazone (CCCP), a known inhibitor of PMF generation that dissipates H^+^ ion gradients. As expected, in the presence of CCCP (>0.1 μM), the persister fraction in the △*msaABCR* reverted to a level comparable to the USA300 LAC and △*msaABCR* + pCN34.*msaABCR* strains (Figure 7B). Likewise, CCCP (10 μM) treatment drastically reduced the membrane potential in both USA300 LAC and △*msaABCR* strains (Figure 7A). The requirement of membrane potential in persister killing is further supported by the results from the individual *ndh*_2_ and *qoxB* mutants. The *ndh*_2_ and *qoxB* mutants showed significantly reduced membrane potential accompanied by an increased persister fraction relative to the isogenic USA300 LAC strain (Figures 7C,D). The *atpA* mutant also showed reduced membraned potential relative to USA300 LAC but was not statistically significant. Additionally, *atpA* mutant did show increased persister fraction and reduced growth rate relative to the USA300 LAC strains (Figures 7C,D).

**FIGURE 8:**
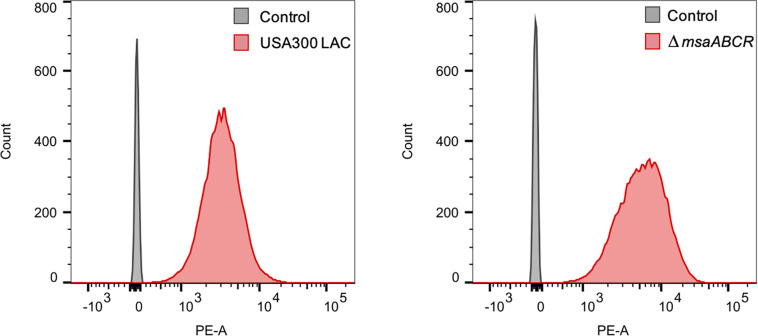
Measurement of gentamicin uptake. The conjugation of gentamicin with Texas Red fluorescence (Gen: TR) in a 30:1 molar ratio was made in anhydrous *N*, *N*-dimethylformamide. The mixture was gently rotated for 3 days at 4°C. The washed stationary-phase cells were incubated with Gen: TR for 3 h at 37°C with continuous shaking. After incubation, the cells were washed twice, resuspended in 500 μl PBS, and evaluated using flow cytometry with excitation/emission maxima at ∼595/615 nm. The median values for PE-A were measured using FlowJo software. The mean of median values for the three independent experiment were 3589 ± 538 for USA300 LAC and 5356 ± 1021 for the △*msaABCR*.

It is known that PMF is necessary for aminoglycoside uptake in bacterial cells, although the complete mechanism underlying this association is not clear (Taber et al., 1987; Allison et al., 2011). Aminoglycoside uptake, in turn, is increased with enhanced membrane potential. Increased membrane potential as well as increased aminoglycoside killing of the △*msaABCR* then led us to examine gentamicin uptake in our test strains using the gentamicin: Texas Red conjugate (Gen: TR) method. Stationary-phase cells were incubated with Gen: TR, and its uptake was measured by flow cytometry. The results showed higher gentamicin uptake in the △*msaABCR* than in the USA300 LAC strain (Figure 8). Together, these results suggest that deletion of *msaABCR* in *S. aureus* increases the membrane potential, which causes higher aminoglycoside uptake, leading to decreased aminoglycoside tolerance.

As we stated above, the stationary-phase △*msaABCR* did not exhibit a low-persister phenotype in response to vancomycin or ciprofloxacin. Therefore, our observations did not sufficiently justify the requirement of ATP for antibiotic tolerance in general, at least in the stationary growth phase. Indeed, a previous study demonstrated that ATP does not determine a persister switch in stationary-phase *S. aureus* cells (Wang et al., 2018). To further investigate this question, we measured ATP concentration in the stationary-phase *ndh*_2_ mutant, which showed a similar level of ATP as the isogenic USA300 LAC strain (Supplementary Figure 1), providing further evidence that ATP does not play a deterministic role in persister generation during stationary growth, especially in the presence of aminoglycoside. Taken together, these observations indicate that membrane potential is required for the aminoglycoside killing of persister cells in the stationary growth phase and that the *msaABCR* operon negatively regulates the generation of membrane potential, thereby contributing to *S. aureus* tolerance toward aminoglycosides.

**FIGURE 9:**
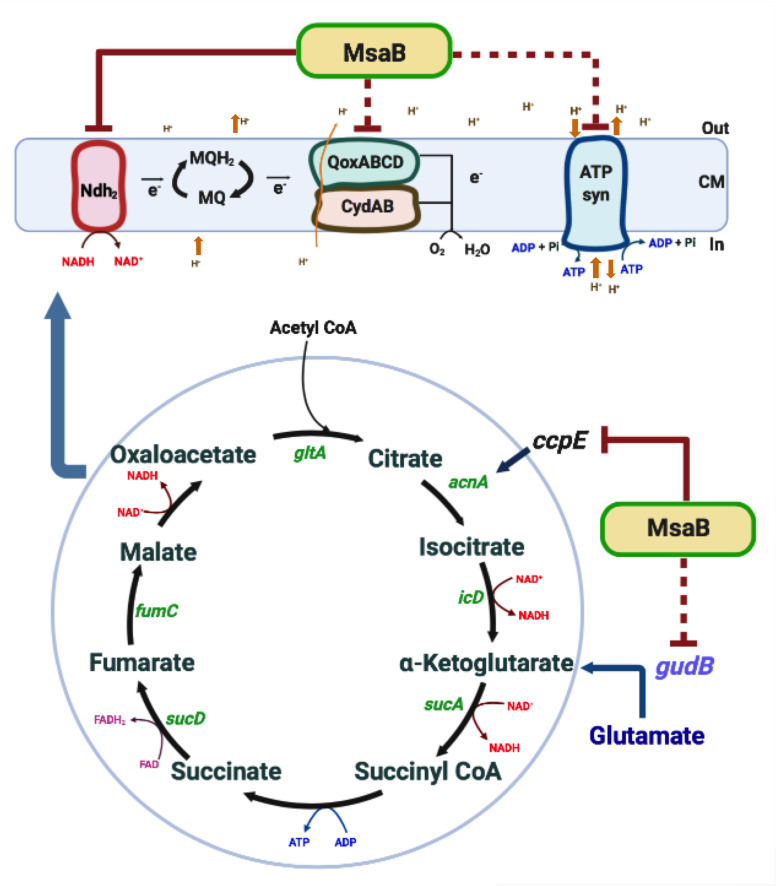
Overview of *msaABCR* (MsaB) regulation of energy metabolism. Inactivation of *msaABCR* in *S. aureus* upregulates *ccpE*, which in turn positively regulates *acnA* expression. The consequent increased activation of the TCA cycle results in increased ATP and NADH. Oxidation of NADH, in turn, increases the membrane potential. MsaB also controls the expression of the ETC gene *ndh*_2_ as an RNA chaperone. This process leads subsequently to the biosynthesis of ATP by the F0F1 ATPase in *S. aureus*.

Based on the observations reported in this study, we propose a mechanistic model of *msaABCR* regulation of TCA cycle activity and NADH oxidation in USA300 LAC cells (Figure 9). The *msaABCR* operon (through MsaB) directly represses *ccpE* and indirectly represses *gudB* to regulate TCA cycle activity and subsequent intracellular ATP levels in *S. aureus* cells. In addition, the *msaABCR* operon (again through MsaB) directly represses *ndh*_2_, a major NADH: quinone oxidoreductase to regulate the generation of membrane potential in *S. aureus* cells.

In conclusion, all the observations reported in this study lead us to conclude that the deletion of the *msaABCR* operon induces a metabolically hyperactive state in *S. aureus* cells, as shown by higher TCA cycle activity and higher ATP content. This increased energy state of the △*msaABCR* leads to decreased persister formation in the presence of different classes of antibiotics in the late exponential growth phase. In stationary phase, however, deletion of the *msaABCR* operon causes increased membrane potential in *S. aureus* cells, which makes a significant contribution to the abrogation of tolerance, specifically toward aminoglycosides. Together, these results suggest different mechanisms of regulation of persister formation by *msaABCR* that are dependent on the growth phase of *S. aureus* cells.

## Discussion

Persister cells, being transiently refractory to antibiotics and able to resuscitate themselves, are strongly associated with recurrent infections displaying clinical significance. Stationary-phase *S. aureus* cells are extremely tolerant to antibiotics, behaving as persister cells (Conlon et al., 2016). Likewise, a stationary-phase subpopulation of *S. aureus* biofilms resembles persister cells, which are largely responsible for treatment failures. Indeed, ample evidence suggests the presence of persister cells in recurrent infections (Conlon, 2014). Therefore, the mechanisms of persister formation as well as the factors affecting their survival and eradication are important matters of investigation that need to be understood to develop effective strategies to control bacterial infections. Lately, these questions have received increasing attention; however, investigating the persister phenotype has been challenging, mostly because of its transience and rarity in *S. aureus* populations. Nonetheless, the general idea about the mechanism of persister formation posits that reduced cellular energy promotes tolerance to antibiotics. In the present study, we attempted to elucidate the *msaABCR* regulation of energy metabolism and its association specifically with aminoglycoside tolerance in stationary-phase *S. aureus* cells.

The killing efficiency of all the bactericidal antibiotics depends on the cellular energy level. Therefore, target inactivation due to a low-energy state should cause increased tolerance to antibiotics in stationary-phase bacterial cells (Lewis, 2007, 2010). Indeed, persister cell formation in a growing staphylococcal population is associated with low cellular energy levels (Conlon et al., 2016; Shan et al., 2017; Cameron et al., 2018), accompanied by low levels of expression of TCA enzymes (Zalis et al., 2019). Although the △*msaABCR* exhibited higher ATP levels, the persister fraction in late exponential phase varied among the antibiotic classes. For instance, gentamicin eradicated △*msaABCR* cells within 48 h. By contrast, a high persister fraction (∼10%) was observed in the presence of ciprofloxacin for more than 4 days, whereas a persister fraction was observed to a lesser extent (∼0.001%) in the presence of vancomycin. While it is not a unique observation that the efficiency of bactericidal antibiotics depends on the energy state of cells, the persister cell phenomenon seems to depend on how the bacterium responds to a stress in general. In support of this speculation, a recent study has indeed demonstrated the formation of intracellular persister cells in response to an antibiotic stress that also displayed multiple stress responses in *S. aureus* (Peyrusson et al., 2020). As we observed a drastic increase in antibiotic tolerance and reduced intracellular ATP levels, our findings confirmed that cellular energy is required for the killing efficiency of bactericidal antibiotics. However, besides ATP, other factors seem to contribute to antibiotic tolerance. These factors may vary, depending largely on the growth phase, which in turn determines whether persister cells are generated or maintained and whether the cells are active or not. For instance, due to slow growth, stationary-phase cells exhibit extreme tolerance to most antibiotics, including vancomycin, which kill actively growing cells. Since the △*msaABCR* has similar growth and yield as that of the USA300 LAC strain, the killing efficiency of vancomycin and ciprofloxacin does not appear to be influenced by ATP concentration, especially in stationary phase. By contrast, we reasoned that the increased energy state along with the presence of active targets contributes to increased killing of the △*msaABCR* by bactericidal antibiotics during late exponential growth phase. It is also possible that deletion of *msaABCR* reduces the stochastic events of reduced TCA enzyme expression during exponential growth phase, resulting in a decreased persister fraction. On the other hand, the fact that stationary-phase △*msaABCR* cells are eradicated only by aminoglycosides suggests a different mechanism of persister killing.

Studies have shown a causal link between persister formation and membrane potential, primarily in the presence of aminoglycosides. A decreased persister fraction was observed in response to enhanced membrane potential and subsequently increased aminoglycoside uptake (Allison et al., 2011; Barraud et al., 2013; Shan et al., 2015). Likewise, reduced membrane potential was shown to cause an increased persister fraction in the TCA mutants of *S. aureus* (Wang et al., 2018). The small-colony variants of *S. aureus* were also found to display increased resistance toward aminoglycosides due to low membrane potential (Kriegeskorte et al., 2014). Likewise, disruption of serine biosynthesis (encoded by *serA*) with subsequently increased PMF led to a decreased persister frequency in *E. coli* (Shan et al., 2015). A recent study showed increased antibiotic tolerance associated with reduced ATP and membrane potential in *S. aureus* cells in a polymicrobial culture (Nabb et al., 2019). Despite a plethora of clues, the complete mechanism linking persister formation and membrane potential is not understood.

According to our observations, it appears that the contributions of the energy state and membrane potential in antibiotic tolerance depend largely on the nature of the antibiotic stress. Nonetheless, we conclude that deletion of *msaABCR* induces *S. aureus* cells to transition to a higher energy state, causing decreased persister cell formation during exponential growth, irrespective of the antibiotics used. Whereas, due to higher membrane potential, stationary-phase △*msaABCR* cells failed to form persister cells, especially in response to aminoglycoside antibiotics.

Considering the urgent clinical relevance of persister cells, understanding the possible mechanisms of their eradication is highly important in selecting a therapeutic target. *S. aureus* systemic infections that are mainly associated with biofilms, which are difficult to treat. So far, eradication of persisters by aminoglycosides has been achieved in conjunction with the metabolites mannitol and fructose, by an ATP-independent acyldepsipeptide antibiotic (ADEP4), by drugs in combination with reactive oxygen species, and by membrane disruption-mediated clearance in different bacterial systems (Allison et al., 2011; Conlon et al., 2013; Briers et al., 2014; Conlon, 2014; Cui et al., 2016). However, there has been a lack of clinical efficacy for these strategies. On the other hand, the use of traditional antibiotics would be beneficial, considering the requirement of exhaustive clinical trials for novel therapeutics.

Administration of gentamicin to treat *S. aureus* infections is widely used in clinical settings, and evidence confirms successful treatment of *S. aureus* systemic and skin infections with gentamicin without any adverse side effects (Richards et al., 1971; Chambers and Pallagrosi, 1973; Buabeng et al., 1999; Hayward et al., 2018). Similarly, treatment with gentamicin was shown to control α-toxin production (Worlitzsch et al., 2001). Likewise, gentamicin was found to shorten treatment duration when prescribed in combination with daptomycin and vancomycin (Tsuji and Rybak, 2005). All these results suggest that gentamicin is a potential antibiotic against *S. aureus* infections. However, increased evidence of treatment failures due to the presence of persister cells warrants identification of the factors influencing their formation. Our observations further indicate that *msaABCR* is a potential target to eradicate *S. aureus* persister cells, and it appears that the use of gentamicin in conjunction with a compound that inhibits *msaABCR* could be a successful therapeutic approach to treating recalcitrant *S. aureus* infections due to the presence of elusive persister cells.

## Data Availability Statement

The raw data supporting the conclusions of this article will be made available by the authors, without undue reservation.

## Author Contributions

All authors designed the project, wrote the manuscript, and read and approved the final manuscript. GS and ME supervised the project. SP and GS performed the experiments.

## Conflict of Interest

The authors declare that the research was conducted in the absence of any commercial or financial relationships that could be construed as a potential conflict of interest.

## Supplementary Material

Supplementary Figure 1 The concentration of ATP in USA300 LAC and the *ndh*_2_ mutant. The data represent the average of three ATP concentrations per CFU of each individual sample measured with two biological replicates. The data represent the results from three independent experiments. Error bars represent the SEM.

Supplementary Table 1 Bacterial strains used in this study.

Supplementary Table 2 Primers used in this study.

Supplementary Table 3 The minimum inhibitory concentration (MIC) of gentamicin, tobramycin, ciprofloxacin, and vancomycin of USA300 LAC, △*msaABCR* and △*msaABCR* + pCN34.*msaABCR* strains.
