# Supplementary material for: The msaABCR Operon Regulates Persister Formation by Modulating Energy Metabolism in Staphylococcus aureus
Source: Front Microbiol. 2021 Apr 14;12:657753. doi: 10.3389/fmicb.2021.657753 (PMC8079656; doi:10.3389/fmicb.2021.657753)
Supplement: Supplementary Figure 1 — The concentration of ATP in USA300 LAC and the ndh2 mutant. The data represent the average of three ATP concentrations per CFU of each individual sample measured with two biological replicates. The data represent the results from three independent experiments. Error bars represent the SEM. [file Data_Sheet_1.docx]

**SUPPLEMENTAL MATERIAL**

**Supplementary Table S1.** Bacterial strains used in this study.

| **Strains** | **Description/Genotype** | **Source** |
| --- | --- | --- |
| *S. aureus* USA300 LAC strain | Methicillin resistant | Dr. Lindsey Shaw |
| △*msaABCR* | *msaABCR* operon deletion mutant in USA300 LAC | (Sahukhal and Elasri, 2014) |
| △*msaABCR*+pCN34.*msaABCR* | *msaABCR* complement in *msaABCR* mutant LAC strain | (Sahukhal and Elasri, 2014) |
| *ndh_2_* | NE 1884 (SAUSA300_0844) | This study |
| *qoxB* | NE 732 (SAUSA300_0962) | This study |
| *atpA* | NE 592 (SAUSA300_2060) | This study |

**Supplementary Table S2.** Primers used in this study.

| **Primers** | **Sequences (5’ 3’)** |
| --- | --- |
| **For qRT-PCR** |  |
| *gltA* F | AAATCGTTATGAAAGAGCAATG |
| *gltA* R | GGATTAGGCTTAAGTGGTTCTT |
| *acnA* F | ATAGTGTTGTAACACCTGAATTAT |
| *acnA* R | CATATAGAGGTTGATCAGTTACAT |
| *icd* F | ATTGCTGACATTTTCTTACAAC |
| *icd* R | AGCATCTGAAATATAGTCACCA |
| *sucA* F | CAGATGACGTTGAAGCTACTAT |
| *sucA* R | CGACGATAACCTACTAAATCAA |
| *sucD* F | TTGACTGAAGAAGGTATTGGTC |
| *sucD* R | TTGACTGAAGAAGGTATTGGTC |
| *sdhA* F | TGAAATTATGACAGCAAATGTAAC |
| *sdhA* R | CAATATCTTCATAACGTTTCATCA |
| *sdhB* F | TTCTATGGTTATCAATGGTCGT |
| *sdhB* R | TAACTGGGAAAGTATTCATTGG |
| *fumC* F | TCGAAGTAGTTTATGGTTTTGC |
| *fumC* R | CGTATACAATGGCATCTTTCTT |
| *ndh_2_* F | AAATATGGTGTGGATCAAAATA |
| *ndh_2_* R | TAACTGCGTGGTTAACTAATTC |
| *qoxB* F | CTTAATAGCTATACAAATTTCAGG |
| *qoxB* R | ATAAACTTCATAGTTGGTGTTTTA |
| *qoxC* F | TACACATGTGGTATTGCTATTTACTA |
| *qoxC* R | CAAAGACTAAACCTAAAAGTAACGTA |
| *qoxD* F | GTTTACGTAACACTATACACGTCA |
| *qoxD* R | TGAACATTAATAATTGAAGTCCTG |
| *atpA* F | TAGAAAGAGCAGCAAAATTAAA |
| *atpA* R | TTGTTGGTACATAAGCTGAAAT |
| *gudB* F | AAGCATTGCATAAATTGGGATT |
| *gudB* R | CCATCATCCATTCGTACAGG |
| **For EMSA** |  |
| *ndh_2_*_EMSA | Biotin-ssDNA-5’-  GTCTTTTTTGTGAACATCACAGGTTTTTACATAATTTAAAAGACTTAAATAACAAAATGATGATAAACTAGTATA |
| *ccpE*_EMSA | Biotin-duplex-5’-  GTTAGTAATGAACTCATGAATGCTTGTGCCATGAAAGTTCAATAATTGAATAATTTATGGGG |
| *gudB*_EMSA | Biotin-duplex-5’- GGAAATTCAATATGTTTGAAAGCGAATCATTAGGTAAAGAAGAGGCGAAAAGGATC |

**Supplementary Table S3.** The minimum inhibitory concentration (MIC) of gentamycin, tobramycin, ciprofloxacin and vancomycin of USA300 LAC, *msaABCR* mutant and complementation strains.

| **Strains** | **Gentamycin (µg/ml)** | **Tobramycin (µg/ml)** | **Vancomycin (µg/ml)** | **Ciprofloxacin (µg/ml)** |
| --- | --- | --- | --- | --- |
| USA300 LAC | 1 | 1 | 0.65 | 2.0 |
| △*msaABCR* | 1 | 1 | 0.325 | 2.0 |
| △*msaABCR*+pCN34.*msaABCR* | 1 | 1 | 0.65 | 2.0 |
| *ndh_2_* | 1 | 1 | 0.65 | 2.0 |
| *qoxB* | 1 | 1 | 0.65 | 2.0 |
| *atpA* | 1 | 1 | 0.65 | 2.0 |

**Supplementary Figure S1.**


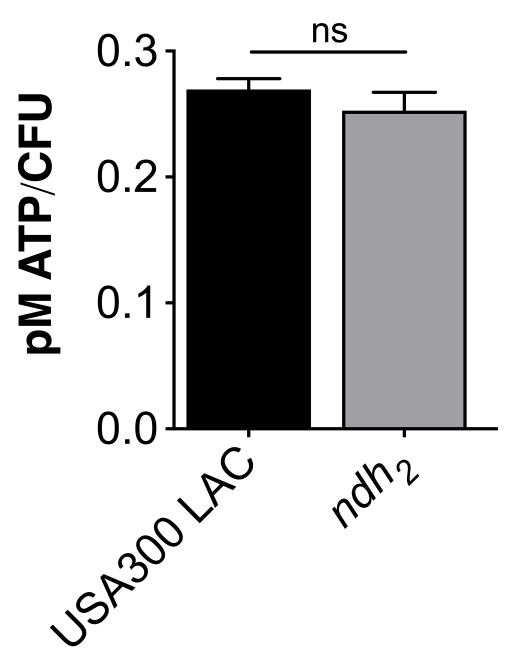


**Figure S1**. The concentration of ATP in USA300 LAC and the *ndh_2_* mutant. The data represent the average of three ATP concentrations per CFU of each individual sample measured with two biological replicates. The data represent the results from three independent experiments. Error bars represent the SEM.

**Supplementary Figure S2**.

**Figure S2.** Full EMSA gel image to examine the binding of MsaB to *ccpE*

**Supplementary Figure S3**.

**Figure S3.** Full EMSA gel image to examine the binding of MsaB to *gudB*

**Supplementary Figure S4**.

**Figure S4.** Full EMSA gel image to examine the binding of MsaB to *ndh_2_*

**REFERENCES**

Sahukhal, G.S., and Elasri, M.O. (2014). Identification and characterization of an operon, *msaABCR*, that controls virulence and biofilm development in *Staphylococcus aureus*. *BMC Microbiol* 14**,** 154.
